# Molecular, Histopathological, and Immunohistochemical Investigation of Peste des Petits Ruminants in Clinically Suspected Sheep in Wasit Province, Iraq

**DOI:** 10.1155/vmi/2702787

**Published:** 2025-07-16

**Authors:** Sattar J. J. Al-Shaeli, Ahmed Jassim Almialy, Ali M. Ethaeb, Hasanain A. J. Gharban

**Affiliations:** ^1^Department of Medical Basic Sciences, College of Dentistry, University of Wasit, Wasit, Iraq; ^2^Department of Veterinary Clinical Sciences, Faculty of Veterinary Medicine, University of Kufa, Najaf, Iraq; ^3^Department of Anatomy and Histology, College of Veterinary Medicine, University of Wasit, Wasit, Iraq; ^4^Department of Internal and Preventive Veterinary Medicine, College of Veterinary Medicine, University of Wasit, Wasit, Iraq

**Keywords:** ELISA, Iraq, *Morbillivirus*, phylogenetic analysis, respiratory diseases

## Abstract

**Background and Aim:** Peste des Petits Ruminants (PPR) has recently become one of the most incidence and threatening viral diseases of sheep in Iraq, which affected notion economy through prevalence and mortality rates. Although there is wide availability of global data on epidemiology, diagnosis, pathogenesis, and treatment of this disease, crucial specific available information with extended prevalence in Iraq still requires specific research to cope. Therefore, this study investigates molecular phylogeny of PPR in sheep during outbreak and detection the histological and immunohistochemical changes in the lung and intestine tissue.

**Materials and Methods:** A total of 182 sheep enrolled in this study that received to the private and official slaughterhouses in Wasit province (Iraq). The sheep examined clinically and subjected to sampling of venous blood that was tested serologically using the enzyme-linked immunosorbent assay (ELISA) and molecularly by polymerase chain reaction (PCR). Postslaughtering, tissue samples were obtained from lung and intestine for histology and immunohistochemistry detection. Phylogenic tree was build based on sequenced of some positive isolates which submitted to the NCBI-database and analysed phylogenetically.

**Results:** Clinically, variable signs were identified in various sheep. The serological anti-PPR IgG antibodies identified 43.41% positive sheep that sequentially allied to mild, moderate and severe PPR cases included 53.16%, 27.85%, and 18.99%, respectively. Molecularly, 24.18% of sheep were infected with PPR, and distinct phylogenetic analysis displayed a close-relationship of study isolates to NCBI-BLAST Mali isolate (MT072487.1). The intestine showed atrophied villi, necrotic mucosal glands, infiltration of lymphocytes and plasma cells in lamina propria, marked diffuse edema, crypt disruption, and cellular necrosis; whereas in lung, macrophages invading alveolar wall, accumulation of inclusion bodies in intracellular and intracytoplasmic regions, infiltration of neutrophils and multinuclear giant cells, and existence of fibrin exudates were seen. Intensity of reaction was relatively ranged from mild (+) to moderate (++) in the intestine, while it was mild (+) in the lung.

**Conclusion:** The displayed results represent the unique Iraqi study that targeted sheep PPR serologically and molecular phylogeny, concomitant with identification of histological and immunohistochemical alteration in the intestine and lung tissues. Therefore, further in depth qualitative and quantitative serological, molecular, and immunological studies focuses on relation of disease with sheep and goats are interesting to identify the prevalence rate, economic losses, and support the control and eradication efforts in Iraq.

## 1. Introduction

Peste des Petits Ruminants (PPR), also known as Ovine Rinderpest, is extremely transmittable viral disease which caused by the *Morbillivirus* genus of the Paramyxoviridae family of the *Orthornavirae* kingdom [1]. Côte d'Ivoire was first to describe the disease in 1942 [2] and since that it has been detected in wide spectrum regions in Australia, South America, Asia, and African countries [3]. Small ruminants including sheep and goats are the targeting animal species affected by the PPR virus; however, camels and a number of wild small ruminants like gazelles and ibex were also affected [4, 5]. The disease is transmitted directly from the infected animals, and indirectly via contact with the contaminated fomites including the ocular and nasal discharges due to presence a large amount of virus in all body secretions and excretions [6, 7].

Fever, cough, nasal and ocular discharges, oral lesions with profound odor, diarrhea, and death within 1-2 weeks are the main clinical symptoms observed in acutely infected sheep; while in subacutely infected cases, signs and lesions are less marked, death is rare and most cases have been recovered [6, 8]. However, severity of infection might be varied based on the proportion of PPR virus from previous exposure, concurrent infections, host breed, and virus strain virulence [9]. For confirmative diagnosis, laboratory tests currently available have been categorized into three groups; virus isolation, detection of viral antigen by specific antibodies using serological detections like ELISA, immunohistochemistry (IHC), and nucleic acid detection using of molecular approach like PCR [10, 11]. Among these assays, PCR represents one of the most important approaches that used to identify and differentiate PPRV from other related viruses due to distinct sensitivity and specificity [12]. Subsequent using of sequencing methods can provide an additional amount of scientific information of potential medical implication at high accuracy, and detection relationship to neighboring strains in other areas or countries [13].

Globally and regionally every single year, PPR disease is involved in massive decline in economy as a result of increasing incidence and death rates in animals associated with dropdown production and elevated cost of treatment and controlling of the disease [14]. According to the Food and Agriculture Organization (FAO), vaccination remains the most effective existed tool to eradicate PPR [15]. However, new clinically outbreaks with various rates of morbidities and mortalities have been observed each year, in particular in last 3 years, in several northern and southern Iraqi areas. Nonetheless, the extensive incidence of PPR in Iraq is acknowledged, and in spite of that, it requires further specific investigation to accurately diagnosis the regional endemic strain and construct prospective incidence map and develop control measurement. Hence, this study was carried out to identify and confirm diagnosis of PPR in clinically suspected sheep through using serological and molecular detection during disease outbreak and identify the sensitivity of diagnostic methodology. Furthermore, the phylogenic analysis of local strains in relation with other global strains was documented in the National Centre for the Biotechnology Information (NCBI). Moreover, identification of histological and immunohistochemical alterations of lung and intestine tissues of infected sheep was detected.

## 2. Materials and Methods

### 2.1. Ethical Approval

This current study was licensed by the Scientific Committee of the College of Veterinary Medicine in the University of Wasit under license number (Ref. No. 139-2592021).

### 2.2. Samples

Throughout the outbreak of PPR disease in Iraq, a total of 182 sheep were brought to the private and official slaughterhouses of Wasit province (Iraq) during October (2021)–March (2022), all of which enrolled in this current study. Before slaughter, each single animal was examined clinically by expert official veterinary doctor to identify the suspected symptoms of PPR. Subsequently, 5 mL of venous blood was drained from animals and divided equally into two parts; one into an EDTA plastic tube for molecular assaying, and the second into free-anticoagulant glass-gel tube that was centrifuged (5000 rpm/5 min) to obtain the sera for serology detection. Both the whole blood and sera were kept frozen at (−20°C) until required for examinations. Furthermore, the lung and intestine tissue samples were collected from some slaughtered animals, and preserved into plastic containers containing 10% buffered neutral formalin as fixative at room temperature until preparing the slides for histology and IHC detection.

### 2.3. Exclude and Include Criteria

Due to national outbreak of PPR disease in several Iraqi regions, the study set without excluded and included criteria and all received sheep are enrolled during the specific period in this study.

### 2.4. Strength and Limitation of the Study

The random sampling was adopted for this study to confirm diagnosis of PPR incidence rate including ELISA and PCR as well as immunohistochemical detection. Furthermore, phylogenic analysis of local strains and close relationship with global strains of PPR was confirmed during disease outbreak period. Moreover, histopathological alterations of both lung and intestine tissue were identified clearly. The main limitations of the study are quiet narrow window for collecting samples and limited numbers of sheep that were received to the private and official slaughterhouse due to random slaughtering in unknown places. Furthermore, due to incidence rate of the disease in local sheep, the owners of animals were anxious, and thus cannot provide us with sufficient required information to determine the epidemiology and risk factors which were absent. Moreover, due to all aspect of epidemiology and risk factors with relevant measurements were not considered due to several challenges; therefore, the suggestion for control models of PPR in Iraq was not including in the study and recommendation for specific serological and molecular broad study was included. Also, effort, time-consuming, and limited budget could have a possible role as study limitations.

### 2.5. Serology

The prevalence rate of anti-PPR antibodies in the sera samples of the study animal was determined using SunLong Biotech (China) sheep ELISA Kit. Following the manufacturer protocols (Cat-No: SL00104Sp), the sera and kits' contents were processed qualitatively, and the absorbance were monitored at 450 nm optical density (OD) through using the ELISA Microplate Reader (BioTek, USA). Subsequently, the CUT OFF value was estimated based on equation (Average Negative Control + 0.15) which set at 0.231. Thus, the positive results were identified when the OD ≥ 0.231 CUT OFF.

### 2.6. Molecular Assay

Firstly, tubes of frozen blood were thawed in water bath (37°C), mixed well by vortex, and then, a total of 200 µL of blood were transferred to a 1.5 mL Eppendorf tube. Following the manufacturer instructions of the GENEzol TriRNA Pure Kit (Geneaid, Korea), a total of 600 µL of GENEzol Reagent was added to initiate the steps of RNA extraction. Subsequently, the total extracted RNA concentration and purity were estimated by using the Nanodrop spectrophotometer (Thermo Scientific, UK). GoScript Reverse Transcriptase Kit (Promega, USA) was used to prepare the cDNA at a final volume of 15 µL following kit protocol. Targeting the nucleoprotein (N) gene, one set of primers [(F: 5′-AGG AGA GGT TGC ACC CTA CA-3′) and (R: 5′-TTT GGG ATC GCA GCT CTG AC-3′)] was build according to global NCBI isolate (ID: KT253999.1). According to GoTaq Green Master Mix Kit (Promega, USA) instructions, the final volume of 20 µL of MasterMix tubes reaction were established, and subjected to the Thermal Cycler (BioRad, USA) conditions as following: one cycle of initial denaturation (95°C/5 min); 35 cycles of 95°C/30 s for denaturation, 56°C/30 s for annealing, and 72°C/30 s for extension; and one cycle of 72°C/5 min for final extension. Finally, the PCR products were run in Ethidium Bromide 1.5% agarose-gel electrophoresis at 80 Am and 100 V for 90 min. The obtained positive results were applied under the UV transilluminator (Clinx Science, China) at an approximate product size of 414 bp.

### 2.7. Phylogeny

Positive DNAs of seven samples were selected randomly and sent to the Macrogen Company (South Korea) for sequences. The obtained sequence data were applied to the NCBI-Database to obtain the specific accession numbers for the local PPR virus isolates and analysed phylogenetically using the multiple sequence alignment analysis and phylogenetic tree analysis in the MEGA-11 Software to detect its global homology identity (%) to the NCBI-BLAST PPR virus isolates.

### 2.8. Histopathology

The formalin-fixed and paraffin-embedded (FFPE) procedure was followed to prepare the tissue sections from the lung and intestine samples. Consequently, the tissues sections (thickness ≈ 4-5 μm) were mounted on glass slides and stained according to SYRBIO, Syria instructions with routine Hematoxylin and Eosin stain. The ready covered slides were examined under the objective lens (× 100, × 400) of light microscope (MEIJI, Japan) [16].

### 2.9. IHC

The PPR virus antigen in tissue sections of lung and intestine was detected using indirect immunoperoxidase technique (IPT) [17]. Manufacturer instructions of the Sheep Anti-PPR Primary Antibody (SunLong Biotech, China), Rabbit Anti-Sheep IgG HRP Conjugate (SunLong Biotech, China), and Hematoxylin and Eosin kits were utilized to complete the reaction on the slides that examined under the objective lens (× 100, × 400) of the light microscope (MEIJI, Japan). According to severity of Ab-Ag reaction, the lesions were classified as mild (+), moderate (++) and severe (+++) [18, 19].

### 2.10. Statistical Analysis

The obtained data were applied in the GraphPad prism Software (*version 8.0.2*), the data analysed by implementing One-Way Analysis of Variance (ANOVA), Turkey multiple comparison post hoc, and Kruskal–Wallis test to detect remarkable differences between the obtained results of clinical examination, serology and molecular assay. The results presented as mean (M) only, the significant were set at *p* < 0.05 (^∗^), *p* < 0.01 (^∗∗^), *p* < 0.001 (^∗∗∗^), and *p* < 0.0001 (^∗∗∗∗^), respectively [20].

## 3. Results

Noticeably, the clinical examination of the sheep showed various mixed clinical symptoms including high temperature (43.41%), congestion of mucous membranes (19.78%), nasal discharge (28.02%), tachypnea (12.64%), respiratory depression (6.04%), cough (18.68%), oral lesion (10.44%), and diarrhea (11.54%) as seen in (Table 1). The symptoms that showed significant elevation compared with others were high temperature, nasal discharge, congestion of mucous membrane, and cough.

**Figure 1:**
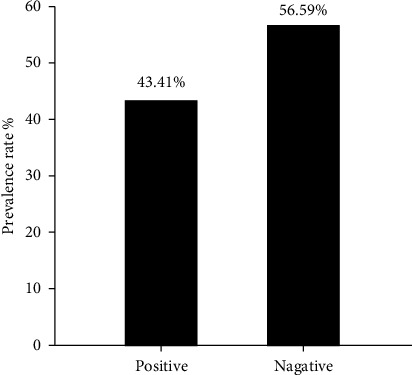
Seroprevalence of anti-PPR IgG antibodies in sheep (*N* = 182).

**Figure 2:**
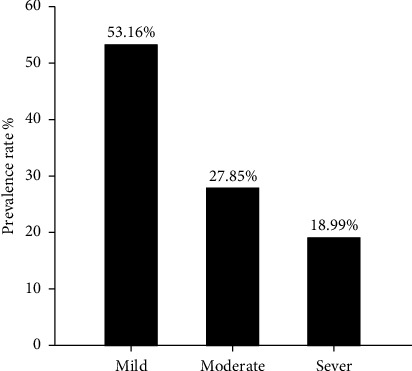
Severity of PPR infection among the ELISA positive sheep (*N* = 79).

**Figure 3:**
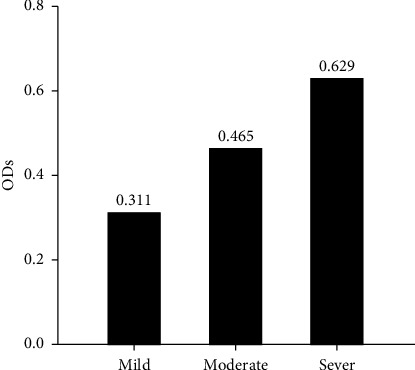
Severity infection and values of ODs in ELISA positive sheep sera (*N* = 79).

Serological detection of anti-PPRV antibodies of 182 sera samples expressed 79 positive samples which represented 43.41% and remaining 103 were negative samples that represented 56.59% as seen in (Figure 1). Crucially, among these positive cases, the significant prevalence rate of mild, moderate and severe PPR were 53.16% (42), 27.85% (22), and 18.99% (15), as displayed in (Figure 2). Subsequently, the recorded values of significant positive ODs were 0.311 ± 0.008, 0.465 ± 0.009, and 0.629 ± 0.013 for mild, moderate, and severe cases, respectively, as seen in (Figure 3).

The findings of clinical symptoms distributed among the positive sheep cases confirmed by ELISA were obviously seen. These mixed symptoms included high temperature (15.19%), congestion of mucous membranes (5.06%), nasal discharge (11.39%), tachypnea (13.92%), respiratory depression (10.13%), cough (20.25%), oral lesion (6.33%), and diarrhea (3.80%) as displayed in (Table 2). The symptoms that showed significant elevation compared with others were cough, high temperature, tachypnea, and nasal discharge.

**Figure 4:**
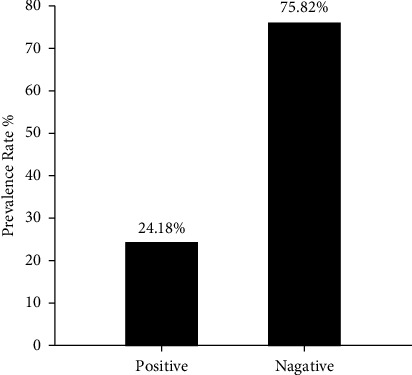
PCR prevalence of PPR in sheep (*N* = 182).

**Figure 5:**
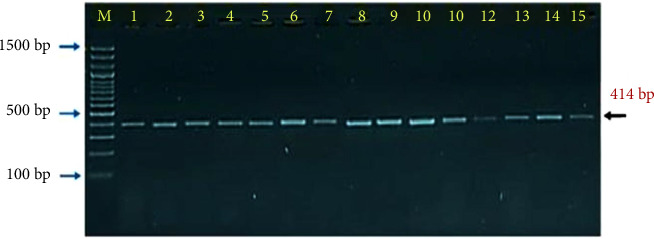
Agarose-gel electrophoresis of PCR products. The PCR product samples run at 80 Am and 100 V for 90 min; in which, Lane (M): Ladder marker (100–1500 bp), Lanes (1–15): representative positive PCR products at 414 bp.

Interestingly, the molecular detection of incidence rate of PPR though targeting the nucleoprotein (N) gene by PCR showed 44 samples positive out of 182 enrolled in the study which represented 24.18% (Figure 4). The PCR products that applied in agarose-gel electrophoresis set on 414 bp for positive samples as seen in (Figure 5).

The clinical symptoms of PCR positive cases were detected to link them with incidence rate of PPR. These clinical symptoms included high temperature (70.45%), congestion of mucous membranes (54.55%), nasal discharge (63.64%), tachypnea (38.64%), respiratory depression (13.64%), cough (38.64%), oral lesion (43.18%), and diarrhea (25.00%) as seen in (Table 3). The symptoms that showed significant elevation compared with others were high temperature, nasal discharge, congestion of mucous membrane, and oral lesion.

**Figure 6:**
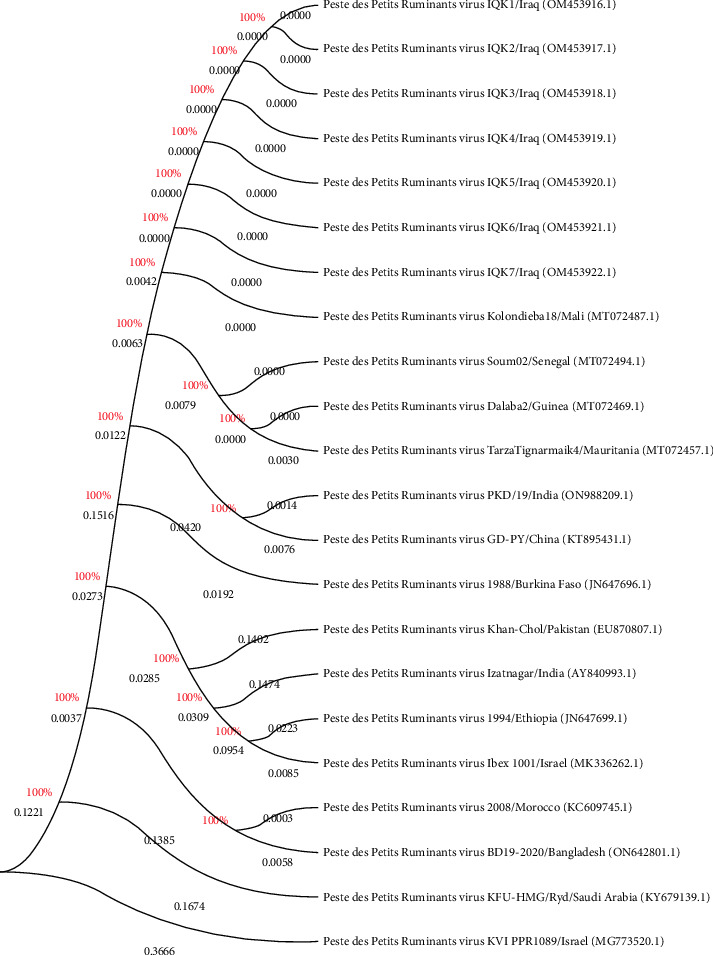
Phylogenetic tree analysis of local and NCBI-BLAST PPR virus isolates/strains.

According to PCR result, sequence data of seven positive DNAs to PPR virus isolates were submitted to the NCBI-GenBank database under the accession numbers of OM453916.1, OM453917.1, OM453918.1, OM453919.1, OM453920.1, OM453921.1, and OM453922.1. Multiple sequence alignment analysis of study local PPR virus isolates with the NCBI-BLAST PPR virus isolates/strains showed the presence of similarity and substitution mutations. Phylogenetic tree analysis displayed a close-relationship between the study local PPR virus isolates with the NCBI-BLAST PPR virus Mali isolate (MT072487.1) at 99.52%–99.55% and total genetic changes at 0.001%–0.005% (Figure 6, Table 4).

**Figure 7:**
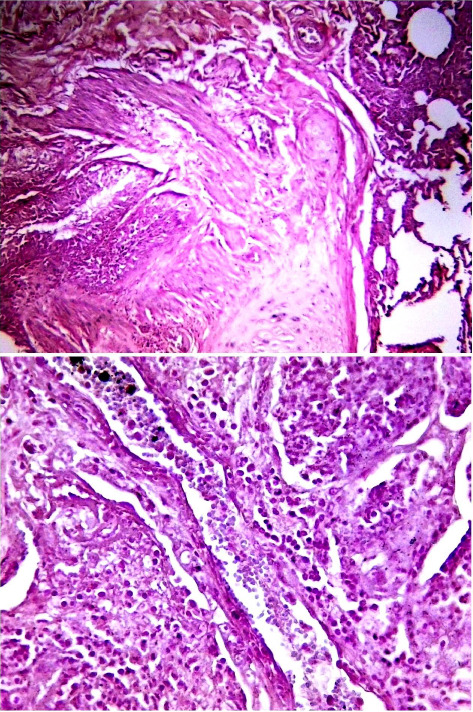
Histological sections of intestine in positive PPR sheep. The histological changes showed atrophied villi, necrotic mucosal glands, infiltration of lymphocytes and plasma cells in lamina propria, marked diffuse of edema, crypt disruption, and cellular necrosis. The image represented the positive samples, and captured at X100 and X400.

**Figure 8:**
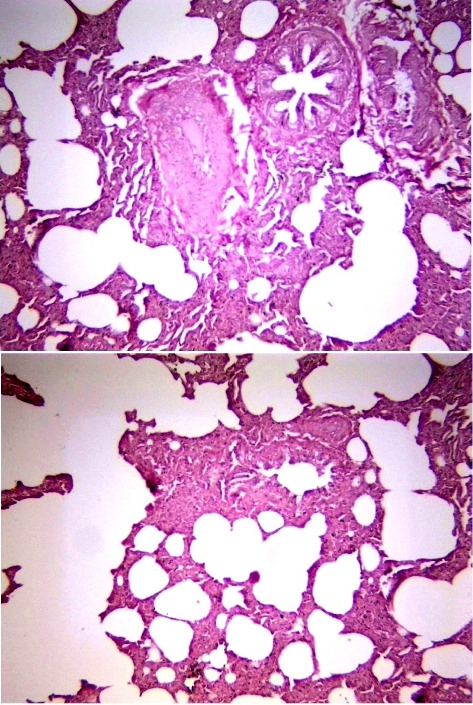
Histological sections of lung in positive PPR sheep. The histological examination showed several tissue changes. The images represented the positive samples and captured at X100 and X400.

Histological examination of intestine tissue sections showed the presence of atrophied villi, necrotic mucosal glands with infiltration of lymphocytes and plasma cells in lamina propria, marked diffuse of edema, crypt disruption, and cellular necrosis in some sections (Figure 7). In lung, infiltration of macrophages, several neutrophils, and multinucleated giant cells in the alveolar regions concomitant with accumulation of inclusion bodies in intracellular and intracytoplasmic region with existing fibrin exudates were seen (Figure 8).

**Figure 9:**
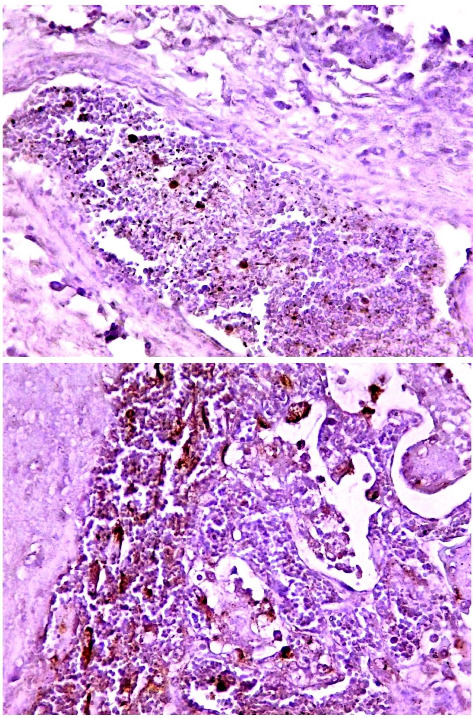
Immunohistochemical detection of PPR antigen in the intestine of positive sheep. The positive reaction of PPR antigen was ranged from mild to moderate based on reaction intensity. The images represent the positive samples and captured at X100 and X400.

**Figure 10:**
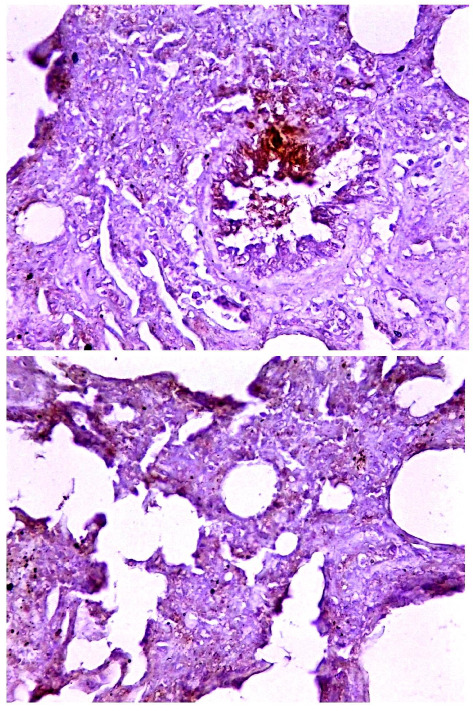
Immunohistochemical detection of PPR antigen in the lung of positive sheep. The positive reaction of PPR antigen was recorded only mild based on reaction intensity. The images represent the positive samples and captured at X100 and X400.

Immunohistochemical examination of intestinal tissue sections identified that the intensity of reaction was relatively ranged from mild (+) to moderate (++) as seen in (Figure 9). Whereas, the lung tissue sections are exhibited only mild (+) reaction as displayed in (Figure 10).

## 4. Discussion

Recent ongoing invasion of PPR to areas unaffected previously has spread widely to several areas in Iraq which generated interest in this research field. In the present study, various clinical symptoms were observed among study sheep, particularly, fever, nasal discharge, and congestion of mucous membrane in addition to other less incidence symptoms included tachypnea, respiratory depression, cough, oral lesion, and diarrhea. Many researchers have been made and developed a clinical scoring method for PPR to determine the intensity of infection in addition to assist the animal owner to euthanized animals that obviously displayed clinical disease [21–24]. Constable et al. [6] mentioned that clinical signs of PPR can be peracute, acute, or subacute, with incidence of elevated body temperature, nasal and ocular discharges, in addition to depression during the first 6 days; 2-3 days later, diarrhea, oral lesion and erosions, dyspnea and cough may appear. The diverse clinical signs of PPR associated with complications resulted from secondary infections can add various range of differential diagnosis which may pose a diagnostic challenge and required laboratory tests to rule out them [6, 25, 26]. In a recent study conducted in Southern region (Iraq), clinical signs of infection were included anorexia (88.6%), erosive oral lesions (77%), dysentery and/or diarrhea (74.6%), weight loss and dehydration (67.3%), ocular discharge and conjunctivitis (57.6%), and respiratory signs (44%) [27]. This variation in prevalence of clinical symptoms might be allied to the impact of the virus specific strain that causes the disease, presence or absence of secondary infections, vaccination programs, and sampling factors (number, region, and season).

In the present study, 43.41% of study sheep were positive to anti-PPR IgG antibodies, from this positive incidence significantly 53.16% was determined as a mild infection in comparison with 27.85% moderate and 18.99% sever. In 1997, the serological study was conducted in central and north areas in Iraq to identify the incidence of PPR in sheep herds. The result showed that the prevalence rate was 21.6% and 30.9% respectively and this data considered the first evidence of PPR incidence in Iraq [28]. One year later exactly in 1998, the Nineveh province recorded the occurrence of official PPR outbreak and the local authorities were responded by rinderpest vaccine [29, 30]. Despite annual compulsory vaccination campaigns from 2011 to 2018, the incidence of PPR was being noticed frequently without any remarkable improvements, in addition to continuous spreading of PPR virus in different regions [31–33]. To compare the current study result of PPR with the findings of other various studies, the seroprevalence of PPR was 51.29% [34] and 61.82% [35] in Pakistan, 0%–52.5% [36] and 2.1%–18.8% [37] in Ethiopia, 10.85% in Algeria [38], 22.41% in Nigeria [39], 63.1% in Sudan [40], 63.8% in Egypt [41], 10%–49.3% in Tanzania [42], 33% in Libya [43], 58% in Iran [44], and 30.91% in India [45]. This variation in PPR seroprevalence in sheep could be due to animal factors including number of animal, gender and age, diagnostic technique employed management practice and ecological aspects of specific demographic region like factors related to sanitary state, settlement directions, climate, and socio-economic status activity [5, 46].

Molecular PCR testing identified that 24.18% was positive to PPR infection. Whereas, serological detection displayed 43.41% of sheep infected with PPR. From serological and molecular obtained data, the latter detected nearly less than half positivity to PPR when compared to serological positivity. Collectively based on these results, the study identified that the molecular testing is more specific, precise, and sensitive technique than the serology to detect PPR virus in sheep. Therefore, the current study suggested using specific PCR test to determine the presence of particular virus in infected animals precisely. Phylogenetic analysis demonstrated that the study of local PPR virus isolates were exhibited a close-relationship to the NCBI-BLAST PPR virus of Mali isolate (MT072487.1). In recent studies in Iraq, the findings revealed that the PPR in sheep of Southern region was related to India isolate (GU014574.1) with whole genetic variation of 0.02%–0.01% [27]; whereas in Al-Diwanyah province, Mansour et al. [47] manifested that the identified strain was closely related to Nigerian strain (MN271586.1). Based on the design and results of these recent close studies [27, 47], the current study is crucially distinct in terms of study period during outbreak, methodology through using serology and molecular detection with immunohistochemical identification, distinct phylogenic analysis and registered strain similarity with NCBI-BLAST PPR virus in 2022, and detection of virus in specific location in Wasit Province. Precisely, the molecular determination of viruses like PPR virus can provide essential and crucial transboundary epidemiological pathways. Furthermore, implemented molecular investigation technique in PPR epidemiology is satisfactory global recommended strategy that provided sufficient knowledge to understand how the PPR is spreading in various countries in order to manage and eliminate it [2]. Fundamentally, understanding the epidemiology and spreading pattern of the PPR virus can achieve through construction phylogenic trees using obtained sequencing and PCR data to describe sequencing isolates to various global lineages [26, 48]. In line with the results of above mentioned studies, the current study findings suggested that various strains of PPR virus might be circulated within sheep flocks in Iraq. Thus, wide molecular study investigate PPR in wide spread regions is particularly of interest to determine the various strains exhibited in Iraq and their origins. Furthermore, construction of incidence map along the risk factors could potentially develop effective management strategy to control PPR infection in various ruminant species in Iraq.

Histological and immunohistochemical assessments of intestine and lung tissue sections were exhibited the common mild to moderate characteristics of infection, that obviously related to disease progress, and similar with that recorded by other researchers [11, 17, 24, 49]. Other studies confirmed that the intestinal PPRV antigens localization with signs and lesions of cellular degeneration and necrosis were similar to those seen in other *Morbillivirus* infections such as rinderpest and canine distemper [17, 25]. Kumar et al. [17] identified that the PPR virus specifically targeting lungs and thus considered the main PPR target organ which could be regarded as a histologically distinguishing feature between PPR and other *Morbillivirus* infections in endemic areas. Parida et al. [11] stated that the intensity of the PPR in infected animals is crucially reflected to the intestinal abnormalities and histological alteration, while histological changes in lung may results from secondary bacterial or parasitic infections. Alwan and Al Saad [27] identified several pathological changes in gastrointestinal tract including intestinal villi stunting and blunting associated with congestion and mucosal desquamation, lamina propria inflammatory mononuclear cells infiltration, submucosal edema, goblet cell hyperplasia, and rarely epithelial syncytia.

## 5. Conclusion

Variations in observed clinical symptoms concomitant with the results of serology and molecular assays indicated that the clinical examination was unable to describe the status of sheep infected with PPR, and the possibility of incidence of secondary infections. This study is considered unique conducted research in Iraq as it utilized both serological and more specific and sensitive molecular methods for detection the PPR infection, and submitted the local isolates in the NCBI database for phylogenetic analysis and its relationship to NCBI-BLAST PPR virus isolates. Furthermore, the study remarkably identified the effect of PPR on intestine and lung histological alterations associated with immunohistochemical detection of virus localization for the first time in Iraq. Fundamentally, to eliminate the disease from all regions in Iraq, clear, and specific map of PPR virus epidemiology required to be built at the national level and this could be achieved through precise extensive qualitative research.

## Figures and Tables

**Table 1:** Symptoms of clinical examination of sheep (*N* = 182).

Symptom	Incidence	
	No.	%
High temperature	79	43.41^∗∗∗∗^
Congestion of mucous membranes	36	19.78^∗∗∗∗^
Nasal discharge	51	28.02^∗∗∗∗^
Tachypnea	23	12.64
Respiratory depression	11	6.04
Cough	34	18.68^∗∗∗∗^
Oral lesion	19	10.44
Diarrhea	21	11.54

*p* value	< 0.0001^∗∗∗∗^	

^∗∗∗∗^Means the *p* value more than 0.0001.

**Table 2:** Symptoms of clinical examination among the ELISA positive sheep (*N* = 79).

Symptom	Incidence	
	No.	%
High temperature	12	15.19^∗∗∗∗^
Congestion of mucous membranes	4	5.06
Nasal discharge	9	11.39^∗∗∗∗^
Tachypnea	11	13.92^∗∗∗∗^
Respiratory depression	8	10.13
Cough	16	20.25^∗∗∗∗^
Oral lesion	5	6.33
Diarrhea	3	3.80

*p* value	< 0.0001^∗∗∗∗^	

^∗∗∗∗^Means the *p* value more than 0.0001.

**Table 3:** Symptoms of clinical examination among the PCR positive sheep (*N* = 44).

Symptom	Incidence	
	No.	%
High temperature	31	70.45^∗∗∗∗^
Congestion of mucous membranes	24	54.55^∗∗∗∗^
Nasal discharge	28	63.64^∗∗∗∗^
Tachypnea	17	38.64
Respiratory depression	6	13.64
Cough	17	38.64
Oral lesion	19	43.18^∗∗∗∗^
Diarrhea	11	25.00

*p* value	< 0.0001^∗∗∗∗^	

^∗∗∗∗^Means the *p* value more than 0.0001.

**Table 4:** Homology sequence identity of the local and NCBI-BLAST PPR virus isolates.

Local PPR virus isolate		NCBI-BLAST PPR virus isolate			
Name	Access no.	Country	Access no.	Host	Identity (%)
IQK.No.1	OM453916.1	Mali	MT072487.1	Goat	99.52
IQK.No.2	OM453917.1	Mali	MT072488.1	Goat	99.55
IQK.No.3	OM453918.1	Mali	MT072488.1	Goat	99.52
IQK.No.4	OM453919.1	Mali	MT072488.1	Goat	99.54
IQK.No.5	OM453920.1	Mali	MT072488.1	Goat	99.55
IQK.No.6	OM453921.1	Mali	MT072488.1	Goat	99.55
IQK.No.7	OM453922.1	Mali	MT072488.1	Goat	99.76

## Data Availability

All obtained and analyzed data in this study are including in this manuscript.
